# The Evaluation of a Modified Dufourmentel Flap after S-Type Excision for Pilonidal Sinus Disease

**DOI:** 10.1155/2013/459147

**Published:** 2013-06-17

**Authors:** Murat Yildar, Faruk Cavdar, Mehmet Kamil Yildiz

**Affiliations:** ^1^Department of General Surgery, Balıkesir University Medical School, Balıkesir, Turkey; ^2^Department of General Surgery, Yalova State Hospital, Yalova, Turkey; ^3^Department of General Surgery, Haydarpaşa Numune Training and Research Hospital, İstanbul, Turkey

## Abstract

*Purpose*. The use of an S-type oblique excision with a bilateral gluteus maximus advancement flap has recently been described for the surgical treatment of sacrococcygeal pilonidal sinus (SPS). Its use in wide lesions has been limited due to the need for a full-thickness flap. We describe the use of an S-type oblique incision together with the Dufourmentel flap in wide lesions. *Method*. Twenty-one patients were treated using a technique including an S-shaped oblique excision of the sinus tract and a broad-pedicled full-thickness flap resembling a Dufourmentel flap to close the defect. *Results*. Of the 21 patients, 19 (90.5%) were male and 2 (9.5%) were female. Their mean age was 24.0 ± 6.1 (range 15–36) years. The mean follow-up period was 14.0 ± 5.8 (range 6–23) months. The postoperative complication rate was 4.8% (one patient), and recurrence was seen in one patient (4.8%). The mean return-to-work time was 13.5 ± 1.9 (range 10–18) days. None of the patients reported dissatisfaction with the cosmetic results. *Conclusions*. This new technique achieved low morbidity and recurrence rates. We anticipate that this will become an important technique in the surgical treatment of SPS if the observed success is confirmed by randomized prospective trials.

## 1. Introduction

A pilonidal sinus is a recurring chronic inflammatory process that is thought to develop in an acquired manner. Hair follicles entering the skin are regarded as the main factor in its pathogenesis [1, 2]. Generally, the disease is seen in the natal cleft in the sacrococcygeal region in patients between the ages of 15 and 35 years. It is twice as common in males as in females and can have a negative effect on the quality of life when symptomatic [3].

Various techniques have been described for the surgical treatment of sacrococcygeal pilonidal sinus (SPS). However, there is no consensus regarding the optimal surgical technique. Several authors have maintained that the ideal treatment in pilonidal sinus surgery can be achieved with flattening and lateralization of the natal cleft [2, 4]. The surgical treatment must be short, require brief hospitalization, increase postoperative patient comfort, and have a short wound healing time and low recurrence rates.

Historically, surgical treatment of pilonidal sinus began with excision and marsupialization or excision and primary closure. Today, these methods have been replaced by excision and closure with advancement flap techniques, which have lower recurrence and complication rates and shorter return-to-work times. Nevertheless, these pilonidal sinus surgery techniques still have room for improvement.

Here, we present the results of a novel technique described as a full-thickness rhomboid transpositional flap (modified Dufourmentel flap) with S-type oblique excision designed for the treatment of pilonidal sinus.

## 2. Materials and Methods

The study population consisted of 21 patients with SPS treated by using an S-type oblique excision and modified Dufourmentel flap between August 2010 and July 2012. Patients with recurrent disease, massive gluteal involvement (diseased area more than 5 cm from the intergluteal sulcus), and acute infection were excluded. Each patient was informed about the operation to be performed and gave their written consent. The operations were performed by the first (MY) and second (FC) authors. Age, gender, body mass index (BMI), primary and recurrent disease, duration of the symptoms (pain, abscess history, and chronic purulent discharge), and length of hospitalization were recorded. The patient characteristics are summarized in Table 1. Patients were evaluated in terms of operation time, postoperative complications (including infection, flap edema, wound dehiscence, seroma formation, flap necrosis, and maceration), recurrence rate, return-to-work time, and cosmetic satisfaction.

### 2.1. Surgical Technique and Postoperative Care

**Figure 1:**
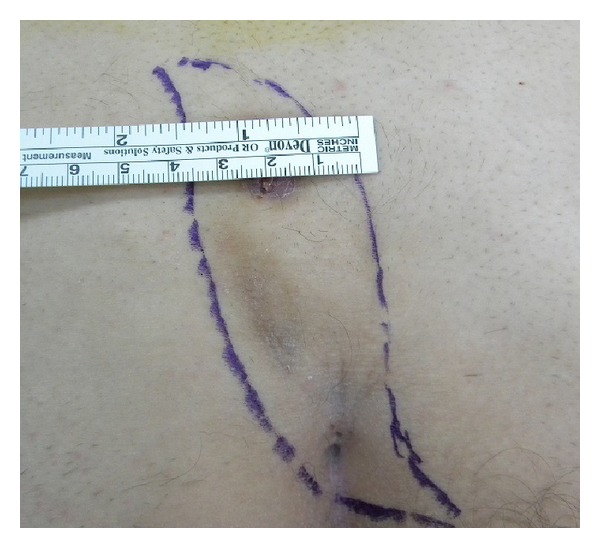
Margins for S-shaped oblique excision including the pilonidal sinus.

**Figure 2:**
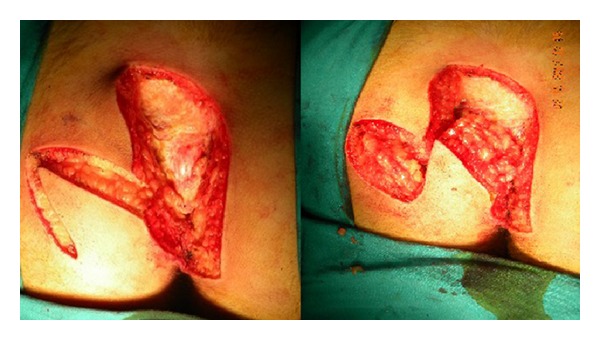
Flap preparation after excision of pilonidal sinus.

**Figure 3:**
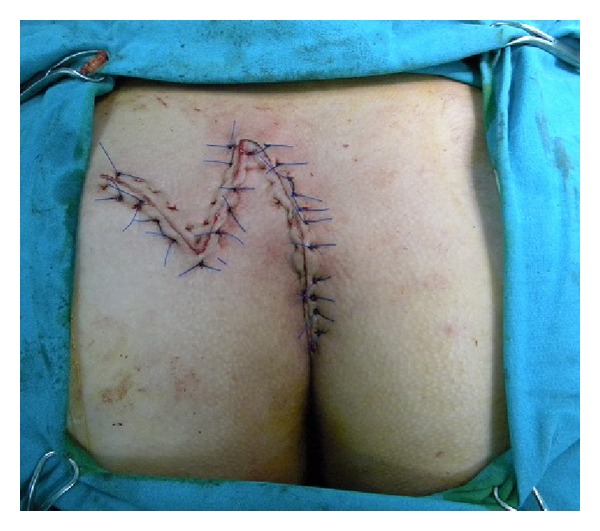
Appearance after flap reconstruction.

In this surgical procedure, the sinus tract was first excised in the prone position, descending as far as the postsacral fascia with an S-shaped oblique incision facing either right or left (Figure 1). A broad-pedicled full-thickness flap resembling a Dufourmentel flap was prepared to include the fascia over the gluteus maximus from the inferior half of the incision (Figure 2). Following careful hemostasis, the flap was transposed onto the defect in the postsacral fascia (Figure 2). The subcutaneous tissue of the flap was sutured to the fascia of the gluteus maximus with polyglactin 0 sutures, and the skin was closed with 2-0 polypropylene sutures (Figure 3). The tissue edges in the area from which the flap was taken were similarly sutured. No drain was used in any patient.

All operations were performed under spinal anesthesia. An enema was administered preoperatively. A single dose of cefazolin (1 g) was administered 30–60 min before the skin incision for prophylaxis.

The patients were mobilized on the first postoperative day and discharged with appropriate instructions for wound care and a 5-day, prescription for oral coamoxicillin (1000 mg) every 12 h. The patients were evaluated on the fifth day, and ultrasonography (USG) was performed to monitor seroma formation. The skin sutures were removed 10–12 days postoperatively. The latest status of patients undergoing surgery was determined by telephone.

## 3. Results

Of the 21 patients 19 (90.5%) were male and 2 (9.5%) were female. The mean patient age was 24.0 ± 6.1 (range 15–36) years. The mean duration of symptoms was 13.0 ± 10.1 (range 3–42) months. The pilonidal sinus was in the chronic phase in all patients. Apart from two cases in which recurrence was identified 2 and 6 months after excision and primary closure in the midline, none of the patients had previously undergone surgery for this disease. The mean BMI for all cases was 25.1 ± 2.8 (range 19.2–29.7) kg/m^2^. The mean operation time was 40.3 ± 4.4 (range 35–50) min. No flap necrosis or wound site infection was seen in any patient postoperatively. A seroma with negative bacterial culture was seen in one patient, and was aspirated. The total complication rate was 4.8% (1/21). The mean return-to-work time was 13.5 ± 1.9 (range 10–18) days, and the mean follow-up was 14.0 ± 5.8 (range 6–23) months. Recurrence was seen in one (4.8%) patient 7 months postoperatively; this patient was treated with excision plus marsupialization. None of the patients reported dissatisfaction regarding the cosmetic results of the surgery.

## 4. Discussion

Pilonidal sinus disease, generally seen in the intergluteal region, was first described by Mayo in 1833 and named by Hodges in 1880 [5].

Techniques such as shaving [6], phenol administration [7], and cryosurgery [8] originally used to treat the disease were found to be inadequate. Historically, the first surgical techniques used to treat pilonidal sinus included lay open, marsupialization, excision, and primary closure. Despite its higher rate of recurrence compared to marsupialization, primary closure postexcision was generally preferred because of its shorter healing time and better patient comfort [9]. Recent studies have shown that the excision used in primary closure affects the recurrence levels [10, 11]. Recurrence is affected by a deep intergluteal sulcus, the effect of vacuum developing between the buttocks, and a midline incision scar [12]. The reported recurrence level with primary midline closure after excision is 20%–42% [9, 13], compared to 0.9–5.6% in primary closure after oblique excision [4, 10].

Flattening/lateralization of the intergluteal sulcus, prevention of seroma formation, reduction of wound tension, and prevention of wound breakdown and scar formation have been suggested to accelerate wound healing and reduce recurrence [4]. Recurrence rates began to decline significantly as advancement flap techniques with these fundamental properties, such as the Limberg flap, began to be used in pilonidal sinus surgery. The Limberg flap technique, first described for the surgical treatment of SPS by Azab et al. [14], is currently one of the most widely used techniques. Topgül et al. [15], Kapan et al. [16], and Akin et al. [17] reported long-term recurrence and early total complication rates in cases treated with Limberg flaps of 2.5% and 6% in 200 patients, 2.3% and 4.6% in 85 patients, and 2.9% and 6.5% in 411 patients, respectively. Although the Limberg flap is a globally accepted technique in the treatment of pilonidal sinus, it was modified by Mentes et al. with translocation of the lower edge neighboring on the anus to 1–1.5 cm lateral to the midline [18]. Using this modification in 198 patients, they reported no recurrence with a mean follow-up of 29.2 ± 3.1 months. These results were supported by subsequent studies. The authors stated that tension-free wound closure was important in the surgical treatment of pilonidal sinus. In a study of 260 patients, Muzi et al. compared tension-free primary closure with the Limberg flap technique and reported no significant difference between the recurrence rates in the two groups (3.8% and 0%, resp.) [19].

**Figure 4:**
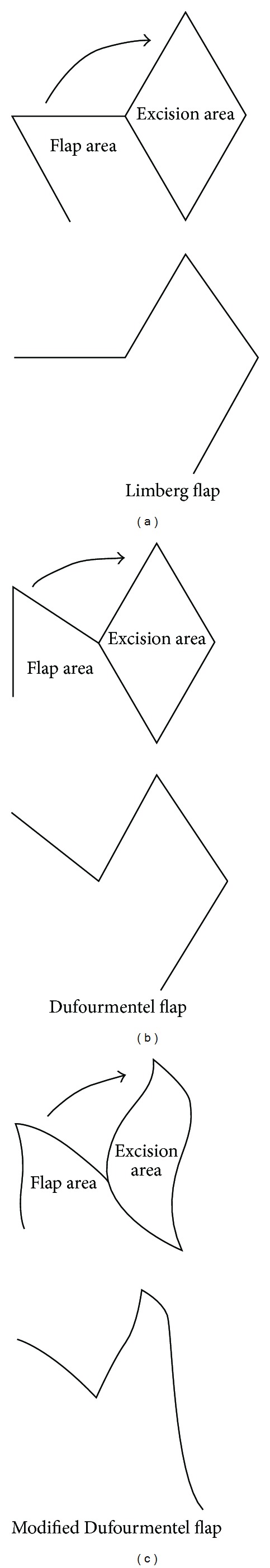
Shows the mapping schemes for the Limberg (a), Dufourmentel (b), and modified Dufourmentel flaps (c) (the final view of the flap procedure is shown at the bottom).

Efforts to reduce recurrence to minimum, lower complication rates and increase patient satisfaction in the treatment of pilonidal sinus are continuing. Recently, the S-type oblique excision for pilonidal sinus was described, and bilateral advancement of the gluteus maximus fascia in addition to primary repair with this excision has been described as a flap reconstruction technique [4, 11]. Krand et al. performed tension-free repair in limited lesions using this technique and reported total complication and recurrence levels of 7.2% and 0.7%, respectively, in a series of 278 patients [4]. In their reconstructions, however, they restricted the area of excision to 3 cm. A literature review of their technique found no data for wider lesions. We believe that this is because their flap technique does not reduce wound tension adequately in wide lesions. The S-type excision can be used only in lesions smaller than 3 cm with a full-thickness flap as described by Krand et al. [4]. We also designed a modified Dufourmentel flap to facilitate application of S-type oblique excision in wide lesions. In our technique, the use of a full-thickness flap in addition to an oblique excision both flattens the intergluteal sulcus and diverts the incision scar from the midline.  Figure 4 shows the mapping schemes for the Limberg, Dufourmentel, and modified Dufourmentel flaps. The operation times were ranged between 40.0 and 57.4 min for full-thickness flap procedures, such as the Limberg and Dufourmentel flaps, while it was 42.8 ± 4.2 min in reconstruction of S-type incisions with the advancement flap described by Krand et al. [4, 20–22]. The operation time was 40.3 ± 4.4 min in our flap technique. The overall complication rate ranges from 0.8% to 25.7% in full-thickness flap procedures, while Krand et al. reported a rate of 7.2% [4, 18, 20, 22]. In the present study, the total complication rate was 4.8%. The recurrence rate ranges from 2.3% to 6.9% with the Limberg flap and was reported to be 2.3% in a study of the Dufourmentel flap procedure by Lieto et al. and 0.7% in a study by Krand et al. [15–17, 21, 22]. We observed a recurrence rate of 4.8%. In addition, our new technique resulted in a mean return-to-work time of 13.5 ± 1.9 (range 10–18) days, which is comparable to that reported in other advancement flap techniques.

## 5. Conclusions

In conclusion, S-type oblique excision used together with the modified Dufourmentel flap procedure can be applied to wider lesions. As it resembles the Limberg flap modification described by Mentes et al. [18], we anticipate that it can reduce the recurrence and complication rates of the classic Dufourmentel flap. If the results are confirmed in future prospective randomized studies, this novel technique with its low morbidity and recurrence rates will become a useful alternative to rhomboid excision used in flap surgery.

## Figures and Tables

**Table 1:** Characteristics of patients.

	Mean ± SEM	Range
Age (years)	24.0 ± 6.1	15–36
Sex (M/F)	19/2 (90.5%/9.5%)	
The mean body mass index (kg/m^2^)	25.1 ± 2.8	19.2–29.7
Patients with recurrent PS (*n*/%)	2/9.5%	
Mean duration symptoms (months)	13.0 ± 10.1	3–42
Pain (*n*/%)	13/61.9%	
Abscess history (*n*/%)	3/14.3%	
Chronic purulent discharge (*n*/%)	5/23.8%	
